# Medical Reports of the Effects of Tobacco, Principally with Regard to Its Diuretic Quality, in the Cure of Dropsies and Dysuries: Together with Some Observations on the Use of Clysters of Tobacco in the Treatment of the Colic

**Published:** 1785

**Authors:** 


III. *Medical Reports of the Effects of Tobacco, principally with regard to its diuretic Quality, in the Cure of Dropsies and Dysuries: Together with some Observations on the Use of Clysters of Tobacco in the Treatment of the Colic. By* Thomas Fowler, M. D. *Physician to the General Infirmary of the County of Stafford.* 8vo. Johnson, *London*, 1785. 84 pages.

SINCE the introduction of Tobacco into Europe, in the year 1560, various medicinal properties have at different times been ascribed to it, particularly by Stahl and other German physicians; but the manner in which of late years it has been spoken of by the generality of writers on the Materia Medica, has occasioned it to be almost wholly excluded from modern practice. This circumstance, however, has not discouraged the author of the work now before us from commencing an inquiry into the medicinal effects of this plant, and the result of his experiments, which are in considerable number, and seem to be accurately and faithfully related, is, that tobacco, under proper regulations, may be administered internally, not only as a safe, but as an efficacious remedy; especially as a powerful diuretic in cases of dropsy and dyfury. This property, amongft the vaft number that have been attributed to it, feems fcarcely to have been hinted at by authors.

The moft ufual form in which Dr. Fowler has given this medicine, is the following, viz.

℞. Foliorum ficcatorum Nicotianæ Virginienfis unciam unam;

Aquæ bullientis libram unam.

Macera per horam unicam in vafe claufo, in Balneo Mariæ pofito, deinde hujus infufi uncias qnatuordecim exprime, et colaturæ adde

Spiritûs vinofi rectificati uncias duas.

Befides this infufion, our author occafionally prefcribes a tincture, a wine and a vinegar of tobacco, prepared by infufing an ounce of the dried leaves of the plant in a pint of rectified fpirit, Spanifh white wine, or vinegar for four days. He likewife mentions a formula for pills, which confifts in mixing a drachm of the leaves in powder, with an equal quantity of conferve of rofes, and as much mucilage of gum arabic as is neceffary to form a mafs, which is to be divided into fixty pills.

Of the infufion he has generally found from fix to an hundred drops, given twice a day in water, cordial julep, or any other proper vehicle, ſufficient to produce the deſired effect in adults ; but in a very irritable patient, he has ſeen twenty-five drops produce ſtronger effects than five hundred did on an old perſon, who had been accuſtomed to tobacco. For a patient of ten years of age, he has commonly found fifty drops ſufficient ; and twenty for one of five. To children under five years of age he has ſeldom ventured to adminiſter it.

The immediate effect of the infuſion is a pungent and tranſient ſenſation of heat in the throat, frequently ſucceeded by a ſenſe of warmth at the ſtomach, as if the patient had taken a ſmall dram. The next general effect of the medicine, taken in a moderate doſe, is diuretic, either with, or without, a ſlight vertigo or giddineſs. In a full doſe, it is more certainly diuretic, often laxative, generally attended with giddineſs, and frequently with nauſea. In painful caſes it proves, in general, anodyne. In ſome it occaſions a drowſineſs, and promotes ſleep ; in others, a drowſineſs, with a ſenſe of heat and reſtleſſneſs.

Dr. Fowler obſerves, that in ninety-three of an hundred and fifteen caſes in which he has given it, it proved diuretic ; that in forty of theſe caſes it occaſioned purging ; that ſeventynine of theſe patients complained of vertigo; and that in fifty-two of the number it excited nauſea. As he has generally found it more or leſs diuretic, in proportion as it has been preceded by more or leſs of vertigo or nauſea, or both, he adviſes us (unleſs the evacuative effect ſhould firſt happen to take place) ſo to increaſe and regulate the doſes, that a vertigo or nauſea may be excited, for the ſpace of thirty or forty minutes. But when theſe effects are in too violent a degree, or a tranſient confuſion of ideas is excited, as he has ſeen happen in two or three caſes, he recommends it to us to ſuſpend the uſe of the medicine till they are abated; and to regulate the future doſes accordingly.

The beſt times for giving the infuſion are ſaid to be two hours before dinner, and at bed time, as it diſagrees moſt with the ſtomach in a morning faſting; and almoſt every patient will, at that time, require to take one fourth, or even one third leſs than in the afternoon.

Of thirty-one caſes of dropſy, in which our author has tried this infuſion, and the particulars of which are related in the work itſelf, eighteen were cured by it, viz. Four of anaſarca, or general dropſy; two of aſcites, and twelve of dropſical ſwellings of the legs. In ten ten other cafes, it afforded confiderable relief, though it did not cure, fo that only three cafes occurred in which it was of no ufe.

Ten inftances of dyfury are related in which the infufion was given, and which prove it to be powerfully anodyne and diuretic in fuch cafes; thereby abating pain, relaxing the urinary paffages, and promoting urine; and in dyfuries arifing from gravel, greatly facilitating the expulfion of calcareous or gritty matter.

The author fpeaks of the ufe of clyfters of tobacco as likely to be of great efficacy in cafes of colic. An ounce of the infufion in half a pint of milk, or common gruel, he thinks, will be fufficient for an adult of an ordinary conftitution; but if this produces no giddinefs or naufea in half an hour, or an hour, (thefe laft effects in cafes of obftinate conftipation moft frequently preceding its laxative operation) we are advifed gradually to increafe the ftrength of the future clyfters, till one or other of thefe effects takes place.

Dr. Fowler has tried the effects of tobacco in a great many difeafes befides dropfies, dyfuries, and colics. In fome eruptive cafes, particularly thofe called fcorbutic, he has employed it with advantage, as he has likewife in afthma, nervous vous head ach, aud hyfterical affections. He has feen a conftant pain of four months ftanding, attending a white fwelling of the elbow in a fcrophulous cafe, entirely removed in eight days by the ufe of the infufion; and he mentions an inftance of tympanites inteftinalis, which after it had refifted, during the fpace of two years, a variety of remedies, was relieved by ftrong clyfters of tobacco.

