# Errata

**Published:** 1785

**Authors:** 


					p. 18(J, 1.16, for from fix to an hundred drops, mi from fixtjr
to an hundred drops.?

				

